# Fabrication of Oil-in-Water Emulsions with Whey Protein Isolate–Puerarin Composites: Environmental Stability and Interfacial Behavior

**DOI:** 10.3390/foods10040705

**Published:** 2021-03-26

**Authors:** Yejun Zhong, Jincheng Zhao, Taotao Dai, Jiangping Ye, Jianyong Wu, Tingting Chen, Chengmei Liu

**Affiliations:** State Key Laboratory of Food Science and Technology, Nanchang University, 235 East Nanjing Road, Nanchang 330047, China; zhongyejun@ncu.edu.cn (Y.Z.); 412314918139@email.ncu.edu.cn (J.Z.); daitaotao@gxaas.net (T.D.); jpye@ncu.edu.cn (J.Y.); jianyongwu@ncu.edu.cn (J.W.); liuchengmei@ncu.edu.cn (C.L.)

**Keywords:** puerarin, polyphenol, whey protein isolates, emulsion, interfacial tension, thermal stability

## Abstract

Protein–polyphenol interactions influence emulsifying properties in both directions. Puerarin (PUE) is an isoflavone that can promote the formation of heat-set gels with whey protein isolate (WPI) through hydrogen bonding. We examined whether PUE improves the emulsifying properties of WPI and the stabilities of the emulsions. We found that forming composites with PUE improves the emulsifying properties of WPI in a concentration-dependent manner. The optimal concentration is 0.5%, which is the highest PUE concentration that can be solubilized in water. The PUE not only decreased the droplet size of the emulsions, but also increased the surface charge by forming composites with the WPI. A 21 day storage test also showed that the maximum PUE concentration improved the emulsion stability the most. A PUE concentration of 0.5% improved the stability of the WPI emulsions against environmental stress, especially thermal treatment. Surface protein loads indicated more protein was adsorbed to the oil droplets, resulting in less interfacial WPI concentration due to an increase in specific surface areas. The use of PUE also decreased the interfacial tension of WPI at the oil–water interface. To conclude, PUE improves the emulsifying activity, storage, and environmental stability of WPI emulsions. This result might be related to the decreased interfacial tension of WPI–PUE composites.

## 1. Introduction

Puerarin (PUE) is the 8-*C*-glucoside of daidzein, an isoflavone that exists in many plants and herbs, notably kudzu (*Pueraria lobata*) root [1]. PUE exhibits anti-inflammatory and antioxidant activities [2]. PUE has broad-spectrum benefits against diseases, including diabetes, cardiovascular disease, cancer, Parkinson’s disease, and Alzheimer’s disease [3]. In addition to these nutraceutical benefits, we previously found that PUE was able to form composites with whey protein isolate (WPI) and promoted its gelation [4]. The interaction of PUE with WPI is mainly through hydrogen bonding. The disulfide bonding formed between WPI might also be influenced by PUE. These interactions altered the conformation of WPI and the aggregation of WPI molecules. However, whether the interaction between WPI and PUE improves the emulsifying properties of WPI was unclear.

WPI is widely used in the food industry as an emulsifier [5,6,7]. It is a nutritious and versatile functional ingredient that is obtained as a byproduct from the dairy industry and consists of β-lactoglobulin, α-lactalbumin, bovine serum albumin, lactoferrin, and immunoglobulin. However, oil droplets stabilized by WPI are particularly sensitive to pH, ionic strength of the aqueous phase, and thermal processing [8]. The processing and storage conditions experienced by the emulsion during its lifetime influence its stability [9]. Therefore, modifications to the WPI have been used to improve the stability of its emulsion, including conjugating with polysaccharides [10], forming nanoparticles [11], etc.

Protein–polyphenol interactions significantly influence the conformation of protein, which leads to changes in its physicochemical and emulsifying properties [12], which depend on the combination of protein and polyphenols. In some cases, an optimum level of polyphenol was required to improve the physical and chemical stability of the protein emulsion, such as tea polyphenols added to whey protein emulsions [7], tannic acid added to pea protein emulsions [13], and phytic acid added to whey protein isolates [14]. Resveratrol was found to disrupt the rice glutelin emulsions and largely increased the oil droplet size [15]. Flaxseed protein isolate emulsion showed higher stability than the emulsion of its protein–polyphenol complex due to higher charge density [16]. No significant influences were found for procyanidin dimer added to rice glutelin emulsions [17]. The polyphenol–protein interaction influences protein characteristics including surface charge, solubility, exposure of sulfide and thiol groups, exposure of hydrophobic groups, etc. These changes collectively determine the emulsifying activity of the protein–polyphenol complex and the ζ-potential, oil droplet size, and susceptibility to oxidation of the emulsions. Environmental factors, such as pH [14], temperature [13], and ionic strength [18], also influence the performance of the emulsion and the effectiveness of polyphenols added to the system. However, the mechanisms through which polyphenols interact with protein and influence its emulsifying activity still need investigation.

In this study, emulsions of WPI only and WPI–PUE composites with different concentrations of PUE were prepared. The interfacial tension in the oil–water interface was measured to understand the influence of PUE on the emulsifying property of WPI. The stability of the emulsions during storage and under environmental stress was also studied. This study provides insight into how the interaction between polyphenol and protein affects the interfacial properties of the protein and the stability of its emulsion.

## 2. Materials and Methods

### 2.1. Material

Puerarin (purity ≥ 97%) was obtained from Aladdin (Shanghai, China). Whey protein isolate (purity 90%) was purchased from Peking Huikangyuan Biotechnology Limited Corporation (Beijing, China). Corn oil was purchased from COFCO (Beijing, China). Nile red was purchased from Sigma (St. Louis, MO, USA). All other chemical reagents were of analytical grade. Purified water with a resistivity of 18.2 MΩ was used to prepare solutions and emulsions.

### 2.2. Preparation of Emulsions

Composites of WPI–PUE were prepared first. The WPI (0.5% *w/v*) was dissolved in water and stirred overnight at 4 °C to ensure full hydration. Then, PUE was added to the WPI solution to reach final concentrations of 0%, 0.1%, 0.2%, 0.3%, 0.4%, and 0.5% (*w/w*). The maximum amount of PUE solubilized in water was 0.5% at room temperature.

Corn oil (10% *w/w*) was added to the WPI–PUE composite solution or the WPI-only solution and then mixed extensively with a high-speed disperser at 12,000 rpm for 3 min to obtain the coarse emulsions. The coarse emulsions were then passed through a high-pressure microfluidizer (Microfluidic M-110EH30, Newton, MA, USA) at 80 MPa three times to obtain the emulsions. To avoid bacterial growth, NaN_3_ (0.02% *w/v*) was added.

### 2.3. Characterizations of the Emulsions

#### 2.3.1. Particle Size

The size and distributions of the emulsion droplets were measured using a static light scattering instrument (Malvern Master Sizer 3000, Worcestershire, U.K.) [19]. To avoid the influence of light scattering caused by dispersant, all emulsions were diluted with either water or solutions with the same pH or ionic strength before measurement. Volume-weighted mean diameters (d_4,3_) were recorded as the mean particle size. The refractive indexes for oil and water were 1.430 and 1.330, respectively, as measured by a refractometer.

#### 2.3.2. ζ-Potential

The ζ-potential was measured using a Zetasizer (Nano-ZSP, Malvern, Worcestershire, U.K.) as described previously [20]. Emulsions were diluted with either water or solutions with the same pH or ionic strength to the appropriate concentration and injected into the folded capillary cell. The ζ-potential value was calculated based on the Henry function.

### 2.4. Confocal Laser Scanning Microscopy

The emulsion was observed using confocal laser scanning microscopy (CLSM, Carl Zeiss LSM710, Jena, Germany) [21]. The emulsion was dyed with 0.1% Nile red (oil phase, ex/em wavelength: 543/605 nm). Then, 100 μL was placed on a glass slide for observation.

### 2.5. Interfacial Protein Concentration

The interfacial protein concentration of the emulsions was measured following the previously described protocol [22]. The emulsion was centrifuged at 13,000 rpm for 30 min to separate the cream layer and serum. The serum in the bottom phase was drawn by a syringe and filtered through a 0.45 μm filter for nonadsorbed protein measurement. The total protein was measured in the original emulsion before centrifuge. The protein concentration was measured by Bradford Protein Assay Kit (Beyotime Biotechnology, Shanghai, China). Adsorbed protein percentage and interfacial protein concentration were calculated using the following equations:

(1) $$\mathrm{Adsorbed}\;\mathrm{protein}\;\mathrm{percentage}\;\%=100\times (W_{total}-W_{\mathrm{serum}})/W_{total}$$

(2) $$Interfacial\;protein\;concentration\;\left(\frac{mg}{m^{2}}\right)=\rho \times d_{32}\times \left(W_{total}-W_{serum}\right)/\left(6\Phi W_{emulsion}\right)$$

Here, *W_total_* and *W_serum_* represent the total protein amount (g) in the emulsion and non-adsorbed protein amount (g) in the serum, respectively; *W_emulsion_* represents the weight of the emulsion (g); *ρ* represents the density of the purified corn oil (0.9292 kg/cm^3^); Φ represents the oil fraction in the emulsions (10% *w/w*); and d32 represents the surface-weighted mean diameter (μm) of the oil droplets.

### 2.6. Dynamic Interfacial Properties

The dynamic interfacial properties of WPI or the WPI–PUE composites at the oil–water interface were analyzed as described previously [23]. Corn oil was purified using florisil absorbents and then placed in the clear glass test cell. The aqueous phase containing WPI or the WPI–PUE composites (13 μL) was injected into the corn oil. The drop shape formed at the tip of the injector was captured and analyzed by a drop shape analyzer (OCA25, Dataphysics Instruments GmbH, Filderstadt, Germany). Measurements were carried out for 8000 s to monitor the kinetics of the composites’ adsorption. The density of the oil and water was 0.9292 and 0.9907 kg/cm^3^, respectively.

### 2.7. Physical Storage Stability

The emulsions stabilized by WPI or the WPI–PUE composites were placed in a tightly sealed serum bottle and stored for 21 days [21]. The visual appearance, particle size, and ζ-potential were recorded at day 0, 1, 3, 5, 7, 14, and 21 during the storage. To avoid bacterial growth, NaN_3_ (0.02%) was added.

### 2.8. Influence of Environmental Conditions on Emulsion Stability

The influence of environmental conditions (pH, salt, and heating) on the properties of emulsions was examined [9]. Particle size and ζ-potential were measured as described in Section 2.3. Visual appearance was recorded using a digital camera.

#### 2.8.1. pH

The influence of different pH values (3, 5, 7, 9, and 11) on the stability of WPI and the WPI–PUE emulsions was assessed. The pH of the emulsions was adjusted with the application of different concentrations of either HCl or NaOH solutions. The visual appearance, particle size, and ζ-potential were measured after being equilibrated overnight.

##### 2.8.2. Ionic Strength

The influence of different ionic strengths on the stability of WPI and the WPI–PUE emulsions was assessed. Emulsions with ionic strength were prepared by mixing the emulsion with different concentrations of NaCl solution to reach the desired ionic strength (0, 100, 200, 300, 400, and 500 mM). The visual appearance, particle size, and ζ-potential were measured after being equilibrated overnight.

##### 2.8.3. Heat

The influence of different temperatures on the stability of WPI and the WPI–PUE emulsions was assessed. The emulsions were incubated at 30, 50, 70, and 90 °C for 60 min and immediately cooled to room temperature. The visual appearance, particle size and ζ-potential were measured after being equilibrated overnight.

### 2.9. Statistical Analysis

The statistical analyses were performed using SPSS (version 22.0, IBM Corp, Armonk, NY, USA). Significant differences (*p* < 0.05) were determined using one-way analysis of variance (ANOVA) followed by Tukey’s post hoc test. All experiments were carried out in triplicates. The values are expressed as mean ± standard deviation.

## 3. Results and Discussion

### 3.1. Characterization of Oil-in-Water Emulsions Prepared Using WPI–PUE Composites

**Figure 1:**
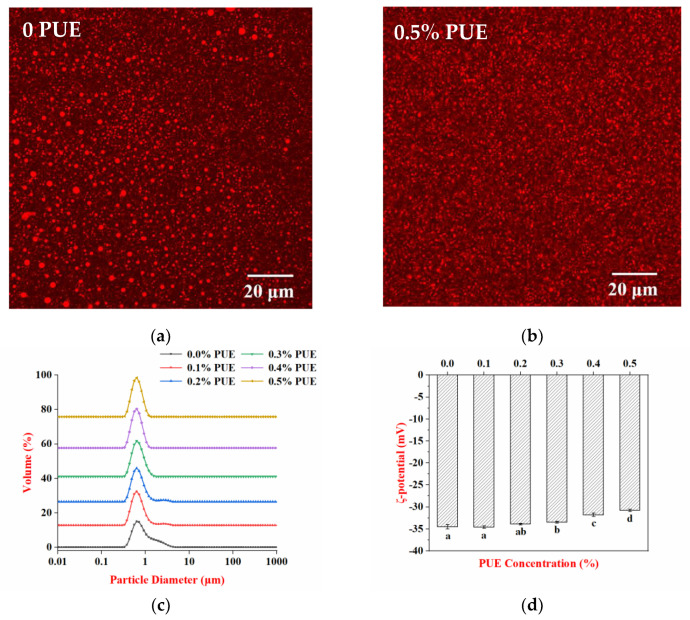
Properties of emulsions stabilized using whey protein isolate (WPI) only (0 puerarin (PUE)) or WPI–PUE composites with different concentrations of PUE (from 0.1% to 0.5%). (**a**) Confocal micrographs of WPI emulsion; (**b**) confocal micrographs of WPI–PUE emulsion with 0.5% PUE, the scale bar corresponds to 20 µm; (**c**) particle size distribution; and (**d**) ζ-potential.

The physical characteristics of emulsions stabilized by WPI and the WPI–PUE composites with different concentrations of PUE are depicted in Figure 1. Emulsions prepared with WPI or the WPI–PUE composites all showed a homogeneous milky white appearance. For emulsion stabilized by WPI only, oil droplets with different sizes were observed (Figure 1a). For the emulsions stabilized by the WPI–PUE composites with 0.1% and 0.2% PUE, some large droplets were observed (data not shown). Oil droplets were uniformly distributed in emulsions stabilized by the WPI–PUE composites with 0.3%, 0.4%, and 0.5% PUE (Figure 1b). Corresponding to the CLSM images, the droplet sizes showed monomodal distributions for all emulsions (Figure 1c). An additional small peak with droplets larger than 1 μm was observed in the WPI emulsion. These observations suggested that PUE improved the emulsifying activity of WPI and created uniform oil droplets [8].

Emulsions stabilized by WPI or the WPI–PUE composites were all negatively charged; the ζ-potential of the emulsions decreased in absolute values with increasing concentration of PUE (Figure 1d). Many reported emulsions stabilized by protein-polyphenol composites showed increased surface charge compared with emulsions stabilized by protein only [13,16]. However, the PUE decreased the negative charge of WPI in this study. This may be attributed to the PUE altering the secondary and tertiary structure of WPI and the shift in the surface charges of the WPI [24]. Previous molecular docking analysis also showed that PUE not only interacted with the positively charged lysine residue, but also interacted with the negatively charged glutamic acid residue [4]. This may also contribute to the decrease in negative charge [25,26].

### 3.2. Storage Stability of Emulsions

**Figure 2:**
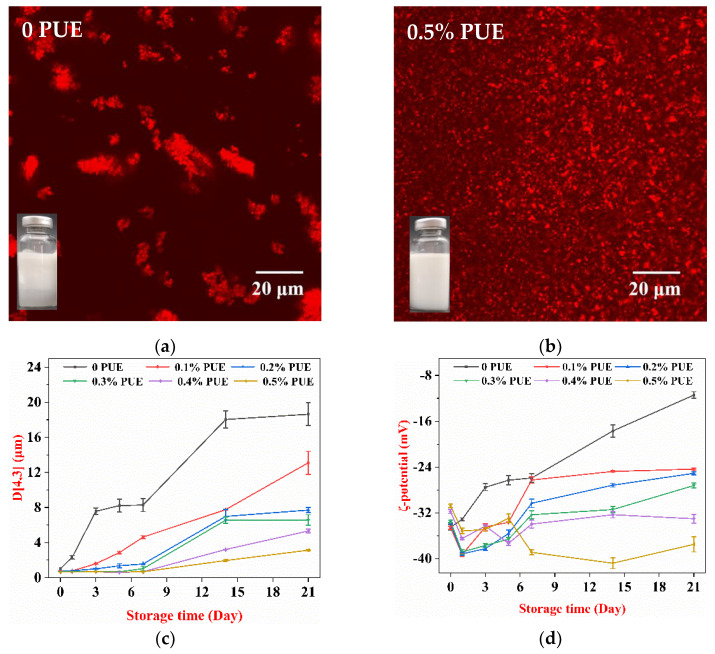
Properties of emulsions stabilized using WPI only (0 PUE) or WPI–PUE composites with different concentrations of PUE (from 0.1% to 0.5%) during 21 days of storage. (**a**) Confocal micrographs of emulsions and visual appearance (bottom left) for WPI emulsion; (**b**) confocal micrographs of emulsions and visual appearance for WPI–PUE emulsion 0.5% PUE, the scale bar corresponds to 20 µm; (**c**) mean particle diameter; and (**d**) ζ-potential.

To evaluate the storage stability and further understand the interactions between WPI and PUE in stabilizing emulsions, the physical property changes of the emulsions were recorded for 21 days (Figure 2). On day 21, phase separation was observed in the WPI emulsion (Figure 2a). No visible change was observed in the appearance of the emulsions stabilized by the WPI–PUE composites with 0.5% PUE. Fewer aggregates were found in the WPI with 0.5% PUE compared with the others (Figure 2b). Larger aggregates were observed in emulsions stabilized by the WPI–PUE composites with less PUE (data not shown). The degree of aggregation increased with decreasing PUE concentration. Corresponding to this, the mean particle size in all emulsions increased over time (Figure 2c). The particle size in the emulsion stabilized by WPI alone increased the most. This result suggested that the WPI–PUE composites with different concentrations of PUE prevent droplet flocculation and creaming during storage in a concentration-dependent manner. The surface activity of WPI was improved by forming composites with PUE [13].

The surface charges of the WPI–PUE emulsions were less than the WPI emulsions and decreased in absolute values with increasing PUE at day 0 (Figure 2d). After one day of storage, the negative charge of emulsion stabilized by the WPI–PUE composites became higher than those stabilized by WPI only. At day 21, the charge of the emulsion stabilized by the WPI–PUE composites with 0.5% PUE remained below −30 mV. The surface charges of other WPI–PUE emulsions all decreased, corresponding to the aggregates observed by CLSM and the dramatically increased droplet size. When WPI adsorbed to the oil–water interface, the molecules shifted from globular form to unfolded form. The nonpolar and sulfhydryl groups were exposed [14,27]. The oil droplet formed flocs by noncovalent bonding or bridging flocculation initially, then stabilized by disulfide bonds between proteins adsorbed to different droplets [28]. Both the initial protein unfolding and the protein crosslinking at the later stage were inhibited when the WPI formed composites with PUE [14], which explained the lower ζ-potential at day 0 and more stable ζ-potential during storage. This inhibition was also observed in other polyphenols. Tea polyphenols have been shown to prevent the aggregation of WPI during storage due to inhibition of oxidation [7].

### 3.3. Influence of Environmental Conditions on the Emulsions

To evaluate the emulsion’s potency in commercial food products, the impact of environmental conditions on the stability of the emulsions prepared using only WPI and the WPI–PUE composites was investigated.

#### 3.3.1. Influence of pH on the Emulsion

**Figure 3:**
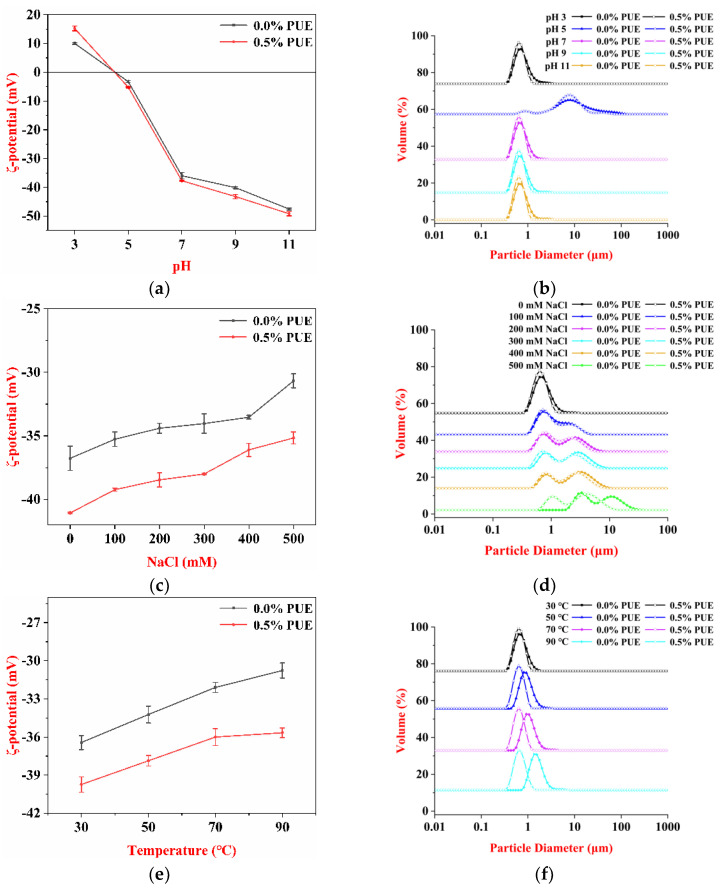
Impact of pH, ionic strength and thermal treatment on the emulsions stabilized by WPI only (0 PUE) or WPI–PUE composites (0.5% PUE). (**a**,**c**,**e**) ζ-potential and (**b**,**d**,**f**) particle size distribution.

In this study, the stability of WPI and the WPI–PUE-stabilized emulsions with pH ranges from 3 to 11 were investigated. Proteins tend to show the minimum surface tension, solubility, conductivity, and stability at the isoelectric point. Therefore, the protein-stabilized emulsion was unstable around its isoelectric point. The isoelectric point of WPI was in the range of pH 4–6 [8]. Therefore, at pH 5, both the WPI and the WPI–PUE emulsions showed extensive phase separation (data not shown). The ζ-potential values were close to zero (Figure 3a). The mean droplet sizes were greatly increased to 14.2 and 8.9 μm for the WPI and the WPI–PUE, respectively (Figure 3b).

For pH below and above the isoelectric point of WPI, no phase separation was observed in the emulsions (data not shown). The ζ-potential was positive for pH 3 and negative for pH 9 and 11 (Figure 3a). With the presence of PUE, the increased absolute ζ-potential indicated that the binding of PUE to WPI increased the electrostatic repulsion of the emulsions in both acidic and alkaline conditions. The strong electrostatic repulsion inhibited the droplet aggregation [29]. The droplet size of the WPI–PUE emulsions was slightly smaller than the corresponding WPI emulsions at the same pH (Figure 3b).

At pH 3, α-lactalbumin was preferentially adsorbed in the cream layer over β-lactoglobulin [30]. β-lactoglobulin presented as monomers, and α-lactalbumin presented as a molten globule with much of the tertiary structure lost at pH 3. PUE interacted with β-lactoglobulin, α-lactalbumin, and other proteins through hydrogen bonding and hydrophobic interactions [4]. These interactions might inhibit the conformational change of WPI proteins at low pH and improve the emulsion stability. At pH 9 and 11, extensive irreversible unfolding and disulfide-mediated polymerization happened to the WPI at room temperature [6]. The presence of PUE might hinder the formation of disulfide bonds and prevent their aggregation or coalescence.

#### 3.3.2. Influence of Ionic Strength on the Emulsion

The presence of salts in the food system could affect the stability of the emulsions involved. Compared with the WPI-stabilized emulsion, the WPI–PUE-stabilized emulsions were more stable against the influence of ionic strength. The macroscopic flocculation was observed in the emulsions with the presence of NaCl starting at 100 mM (data not shown). The negative charges decreased with the increase in ionic strength (Figure 3c). The conformation of WPI was altered in the presence of PUE, which might result in more exposure of negatively charged groups. The negative charges were higher in the WPI–PUE emulsions than in the WPI emulsions. Though all mean particle sizes increased with the increase in NaCl from 0 to 500 mM, the droplet sizes in the WPI–PUE emulsions were all smaller than in the WPI emulsions (Figure 3d).

The addition of salt to the emulsions shielded the electrostatic screening effects, and therefore decreased the electrostatic repulsion [20]. Though the presence of PUE increased the ionic stability, both the WPI and the WPI–PUE emulsions were unstable in the presence of salt. The WPI–PUE composites might also disassemble because of electrostatic shielding in the presence of NaCl [26].

#### 3.3.3. Influence of Thermal Treatment on the Emulsion

The effect of temperature on the emulsion stability was evaluated by heating the samples from 30 to 90 °C. Heat treatment of the WPI emulsions at high temperatures resulted in the loss of stability. Lower negative ζ-potential was observed in the WPI emulsions compared with the WPI–PUE emulsions at all temperatures (Figure 3e). As a result, the static repulsion between the oil droplets increased and improved the stability of the emulsion. The droplet size of the WPI emulsion increased with the temperature (Figure 3f). After heating, the increased droplet size indicated the aggregation or coalescence of oil droplets, but those with the WPI–PUE emulsion remained unchanged. The emulsion stabilized by WPI was relatively unstable against heat [22]. Upon heating, the WPI unfolded and aggregated, which led to the aggregation of the oil droplets and creaming of the emulsion [31]. We suggest that the presence of PUE prevented the unfolding of the WPI and reduced the crosslinking, i.e., disulfide bond, between the WPI molecules [14]. The reduced covalent bonding then prevented the aggregation of the droplets during heat treatment [32].

### 3.4. Adsorption of WPI–PUE Composites at the Oil–Water Interface

**Figure 4:**
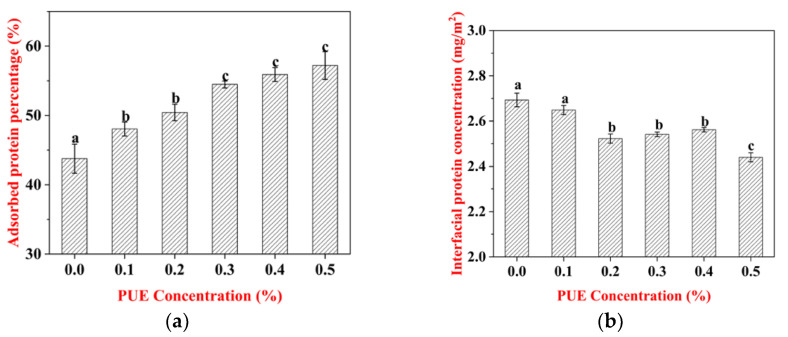
Surface properties of emulsions stabilized by WPI only or WPI–PUE composites with different concentrations of PUE (from 0.1% to 0.5%). (**a**) Interfacial protein concentration and (**b**) adsorbed protein percentage.

The percentage of adsorbed WPI on the oil droplets increased after forming composites with PUE (Figure 4a). The percentage of adsorbed WPI was 43.77% ± 2.1% in the WPI-stabilized emulsion and climbed to 57.21% ± 2.5% with the presence of 0.5% PUE. However, the protein surface load of the WPI–PUE emulsions was reduced compared with the WPI emulsions (Figure 4b). The surface load decreased with increasing concentrations of PUE. Emulsion stabilized by the WPI with 0.5% PUE had the least amount of protein surface (2.44 ± 0.02 mg/m^2^). The competitive adsorption between protein and polyphenol at the oil–water interface could contribute to the decrease in the protein amount [24]. The surface activity of the WPI was improved after forming composites with PUE. As a result, less WPI is required to stabilize a specific oil droplet compared with WPI only. The surface-weighted mean diameters (d3,2) for the emulsion of the WPI-only and the WPI–PUE composites decreased with increasing PUE (data not shown). Therefore, the increased adsorbed protein and the reduced interfacial concentration in WPI–PUE emulsions were considered to be the result of the increased number and reduced size of the oil droplets.

### 3.5. Interfacial Tension of WPI–PUE Composites at the Oil–Water Interface

**Figure 5:**
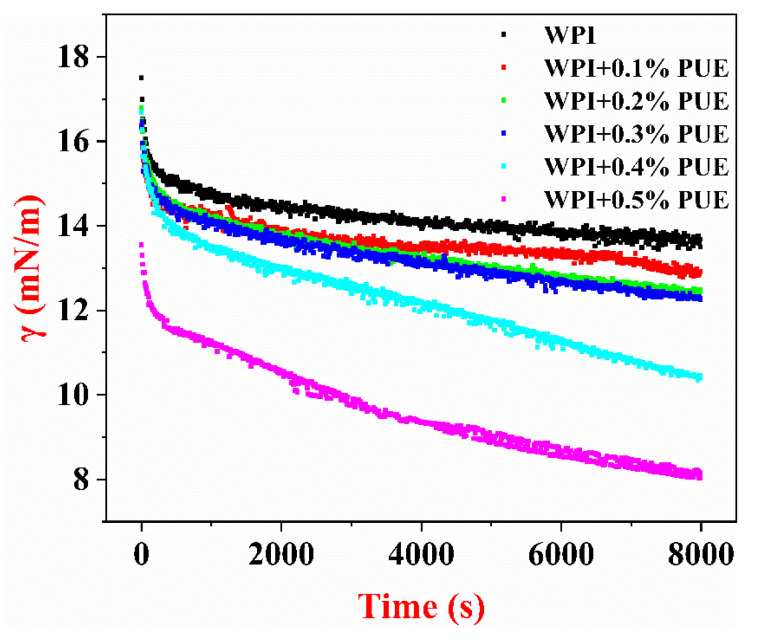
Dynamic interfacial tension (γ) of WPI only or the WPI–PUE composites at the oil–water interface with increasing adsorption time.

To further understand the effects of forming composites with PUE on the surface activity of WPI, the interfacial tension (γ) of WPI or the WPI–PUE composites at the oil–water interface was studied (Figure 5). It is critical to minimize the interfacial tension between continuous and dispersed phases in emulsions to enhance the stability against its immiscible nature [24]. A typical dynamic interfacial tension curve of a protein emulsifier consists of two phases [33]. The initial phase has a steep slope represented by the phase when the protein emulsifier moves toward the oil–water interface and is adsorbed to the interface. At this stage, conformational change and rearranging of the proteins occurs. The second phase, which has a nearly horizontal slope, corresponds to the aging of the proteins at the interface. In this study, the dynamic interfacial tension of WPI and the WPI–PUE composites all exhibited typical changes in protein emulsifiers. The interfacial tension decreased for all samples during the measurement. At the initial phase, WPI and the WPI–PUE at different concentrations other than 0.5% showed similar speed in adsorption to the oil–water interface (Figure 5). The interfacial tension of the WPI–PUE composites after 8000 s of adsorption was lower than that of the WPI alone. The interfacial tension of WPI with 0.5% PUE was significantly lower in both phases, indicating that composition with 0.5% PUE significantly altered the ability of the WPI to be adsorbed around the oil droplets, as well as the conformation of WPI at the oil–water interface. The interaction between WPI and PUE, including the pi stacking between aromatic rings of WPI and PUE, significantly altered the rearrangement of the WPI at the oil–water interface. Thus, the exposure of tryptophan, as indicated by fluorescence emission spectra, and the alteration in the hydrophobic domains were both altered [4,34]. It is suggested that PUE stacked to hydrophobic side chains of the amino acids and inhibited the unfolding of the WPI [35]. As a result, the interfacial tension of the WPI–PUE decreased.

## 4. Conclusions

In this study, we found a novel composite of WPI and PUE. The use of PUE not only improved the surface activity of WPI, but also enhanced the storage and thermal stability of the WPI emulsions. The emulsions stabilized by the WPI–PUE composites with 0.5% PUE were the most stable because of the reduced interfacial tension. By comparing with WPI only, the interfacial tension of WPI significantly reduced after forming composites with PUE. Fewer WPI–PUE composites were required to stabilize the oil droplets with the same surface. More WPI–PUE composites adsorbed to the oil droplets in the whole emulsion as the result of decreased droplet size and more homogeneous emulsion. The biggest advantage of the WPI–PUE emulsion was its stability under thermal treatment. This is attributed to the interference of PUE on the conformation shift of the WPI adsorbed to the oil droplets and the disulfide bond formation of WPI during storage. However, the WPI–PUE composites also showed limitations under ionic strength, and therefore cannot be used in foods with high-salt conditions. The findings of this study not only provide a novel composite for thermal stable emulsions, but also provide information on how to improve the thermal stability of protein emulsifiers.
